# High prevalence of targetable drivers but poor outcomes in lung adenocarcinoma: a real-world cohort from the French West Indies

**DOI:** 10.3389/fonc.2026.1765967

**Published:** 2026-05-04

**Authors:** Régine Marlin, Emeline Colomba, Sabrina Pennont, Adel Zouzou, Marlyne Moranton, Nassim Boukadoum, Heriniaina Randriamiarisoa, Jean-Marc Rakotonarivo, Stefanos Bougas, Karim Fard, Soizic Masson, Moustapha Dramé, Jacques Jougon, Mihaela Aldea, Moustapha Agossou

**Affiliations:** 1 Molecular Biology Department, Centre Hospitalier Universitaire (CHU) Pierre Zobda Quitman, Fort-de-France, Martinique; 2 Medical Oncology Department, Centre Hospitalier Universitaire (CHU) Pierre Zobda Quitman, Fort-de-France, Martinique; 3 Pneumology Department, Centre Hospitalier Universitaire (CHU) Pierre Zobda Quitman, Fort-de-France, Martinique; 4 Radiation Oncology Department, Clarac Hospital, Centre Hospitalier Universitaire (CHU) Pierre Zobda Quitman, Fort-de-France, Martinique; 5 Pathology Department, Centre Hospitalier Universitaire (CHU) Pierre Zobda Quitman, Fort-de-France, Martinique; 6 Medical Oncology Department, Clarac Hospital, Centre Hospitalier Universitaire (CHU) Pierre Zobda Quitman, Fort-de-France, Martinique; 7 Caribbean Institute of Nuclear Imaging (ICIN), Centre Hospitalier Universitaire (CHU) Pierre Zobda Quitman, Fort-de-France, Martinique; 8 Clinical Research Directorate, Functional Unit (UF) 3603, Centre Hospitalier Universitaire (CHU) Pierre Zobda Quitman, Fort-de-France, Martinique; 9 Department of Medical Oncology, Thoracic and Precision Medicine Group, Gustave Roussy Cancer Center, Paris-Saclay University, Villejuif, France

**Keywords:** French West Indies, lung adenocarcinoma, molecular profiling, PD-L1 expression, real-world data, targetable oncogenic drivers

## Abstract

**Introduction:**

Lung adenocarcinoma remains the most common subtype of non–small cell lung cancer (NSCLC), yet molecular epidemiology and real-world outcomes in Caribbean populations are poorly documented.

**Methods:**

We conducted an 18-month, retrospective, population-based study including all consecutive patients diagnosed with lung adenocarcinoma in the French West Indies. Clinical, pathological, molecular, and survival data were collected at the University Hospital of Martinique, the sole referral center on the island.

**Results:**

Of 159 patients, 58.5% presented with *de novo* metastatic disease and 14.4% had an unknown stage, more than half of whom died within 17 days, reflecting substantial diagnostic delays and early mortality. Actionable oncogenic drivers were identified in 62% of tumors, including *EGFR* (33%), *KRAS* (18%), *BRAF* (4%), *ERBB2* exon 20 (4%), *ALK* (3%), *ROS1* (3%), *RET* (1%), *NRG1* (1%), and *MET* exon 14 skipping (4%). PD-L1 expression ≥1% was observed in 61% of patients, with a comparatively low proportion of PD-L1–high tumors, consistent with the high prevalence of *EGFR* mutations. Median overall survival was 19.3 months. Progression-free survival was longer with targeted therapies compared with immunotherapy ± chemotherapy, although overall survival remained similar.

**Discussion:**

This study provides the first integrated clinical and molecular characterization of lung adenocarcinoma in the French West Indies and highlights the urgent need to improve early diagnosis, molecular testing access, and treatment initiation in underserved island populations.

## Introduction

Lung cancer remains the leading cause of cancer-related mortality worldwide, despite major advances in screening, molecular diagnostics, and targeted therapies (1). In France, disparities in access to cancer care according to geographic and socio-economic context are well recognized. Beyond continental Europe, France encompasses several overseas territories located in the Atlantic, Indian and Pacific Oceans, where healthcare delivery is shaped by specific demographic, environmental, and logistical constraints. The epidemiology of cancer in these regions differs from that of mainland France, particularly in territories with predominantly African or mixed ancestry populations, as highlighted in the FRANCIM 2024 report (2). Importantly, these populations remain underrepresented in clinical trials, thereby limiting the generalizability of recent advances in precision oncology (3–5).

In Martinique, an annual average of 89 new lung cancer cases in men and 51 in women were reported between 2007 and 2014, corresponding to approximately 140 cases per year. Lung cancer accounted for 4.1% of incident cancers in men and 3.6% in women, while mortality reached 84 deaths per year in men (9.8% of all cancer-related deaths) and 49 in women (7.4%). Compared with mainland France, both incidence and mortality remain lower, confirming a relative under-incidence. Nevertheless, the burden remains substantial, as lung cancer continues to rank among the leading causes of cancer-related death on the island (2).

Molecular characterization of lung adenocarcinoma has become essential to guide therapeutic decision-making (6, 7). The expanding availability of targeted therapies and immune checkpoint inhibitors has markedly improved outcomes in advanced non–small cell lung cancer (NSCLC) (8–10). However, clinical, pathological, and survival data from Caribbean populations remain scarce, and little is known about the applicability of precision oncology strategies in this context.

Within this setting, the objective of our study was to characterize the molecular profiles of all consecutive cases of lung adenocarcinoma diagnosed in Martinique over an 18-month period, and to assess the contribution of systematic molecular testing to therapeutic management, survival outcomes, and delays in treatment access in this unique and understudied population.

## Materials and methods

### Materials

This retrospective, single-center study included all consecutive patients diagnosed with lung adenocarcinoma in Martinique between January 2023 and July 2024. Martinique is a French Caribbean island with a centralized healthcare system for thoracic oncology. All patients diagnosed with lung cancer across the island are systematically referred to the Department of Pneumology at the University Hospital of Martinique, which serves as the sole referral center for diagnosis, molecular profiling, and treatment initiation. Therefore, despite its monocentric design, this study reflects a population-based cohort encompassing all eligible cases diagnosed within the defined period. The study was approved by the institutional review board (IRB 2023/007) of the University Hospital of Martinique, and data were extracted from internal hospital databases.

Clinical variables included age at diagnosis, sex, smoking status, ECOG performance status, comorbidities, allergies, and occupational exposures, all collected at the time of diagnosis. Smoking status was categorized as never-smoker, former smoker, or current smoker. Individuals reporting passive smoking exposure were classified as never-smokers, in line with international thoracic oncology conventions (11–13). Patients reporting exclusive cannabis use were grouped with smokers due to shared exposure to inhaled combustion products.

### Molecular analysis and PDL1 expression

Molecular analyses were performed on formalin-fixed, paraffin-embedded (FFPE) tumor tissue obtained at diagnosis. DNA profiling was performed using a targeted hotspot panel (Illumina, San Diego, CA) sequenced on the MiSeq platform, and RNA fusion detection employed the Archer FusionPlex assay (ArcherDx, Boulder, CO). This combined DNA and RNA enabled the detection of point mutations, insertions/deletions, exon skipping events, and gene fusions across major NSCLC oncogenic drivers (*EGFR*, *KRAS*, *BRAF*, *ERBB2*, *ALK*, *ROS1*, *RET*, *MET*, *NRG1*).

PD-L1 expression was assessed by immunohistochemistry (IHC) on FFPE tumor sections using an FDA-approved, clinically validated PD-L1 assay. Results were reported as Tumor Proportion Score (TPS) and categorized as <1%, 1–49%, or ≥50% according to current international guidelines.

### Statistical analysis

For survival analyses, overall survival (OS) was defined as the time from diagnosis to death from any cause. Patients who were alive at last follow-up or lost to follow-up were censored at the date of last known contact. Median follow-up time was calculated among patients alive at last contact, in order to reflect the duration of observation among censored individuals (14). Progression-free survival (PFS) was defined as the time from treatment initiation to documented disease progression or death. Patients without documented progression or death at last follow-up were censored at the date of last assessment. Kaplan–Meier methods were used to estimate median OS and PFS with 95% confidence intervals, as well as 6-, 12-, and 24-month survival rates. Comparisons of OS and PFS between treatment groups were unadjusted and exploratory and were not intended to assess causal treatment effects. Given that treatment allocation was strongly influenced by baseline clinical factors—including molecular status, disease stage, and performance status—confounding by indication was considered likely. Consequently, no multivariable survival models were performed, as the sample size and heterogeneity of the cohort would have limited the stability and interpretability of adjusted analyses.

The relationship between PD-L1 expression (TPS categories) and main driver mutation status (*EGFR* or *KRAS*) was assessed using contingency tables. Chi-square tests (χ²) were applied to determine whether PD-L1 distribution differed between mutation-positive and mutation-negative subgroups.

Among patients treated with chemotherapy ± immunotherapy, we evaluated the association between PD-L1 expression and documented disease progression. PD-L1 was analyzed as a categorical variable (<1%, 1–49%, ≥50%), and comparisons across groups were performed using χ² testing. This exploratory analysis aimed to assess whether PD-L1 expression was associated with treatment response in real-world conditions.

Statistical significance was defined as p < 0.05 (two-sided). All analyses were performed using Python (custom scripts) and cross-validated against standard statistical packages.

## Results

### Clinical and demographic characteristics of the cohort

Between January 1, 2023, and July 30, 2024, 159 patients were diagnosed with non-small cell lung adenocarcinoma. The clinical and demographic characteristics of the cohort are listed in **Table 1**. The median age at diagnosis was 67.97 years (range: 42.95–92.15). A history of active or former tobacco use was reported in 47 patients (29.55%), while 2 (1.3%) reported passive exposure and 1 (0.6%) had a transient smoking history. Additionally, 6 patients (3.8%) had documented allergies and 7 (4.4%) reported occupational exposure to potentially harmful agents.

**Table 1:** Clinical and demographic characteristics of the cohort.

Variable	Value
Number of patients	159
Age at diagnosis, median (range), years	67.97 (42.95–92.15)
Sex, n (%)	
Female	72 (45.3)
Male	87 (54.7)
Smoking status, n (%)	
Never smoker	Not available
Current or former smoker	47 (29.55)
Passive exposure	2 (1.3)
Transient smoking history	1 (0.6)
Documented allergies, n (%)	6 (3.8)
Occupational exposure to potentially harmful agents, n (%)	7 (4.4)
Disease stage at diagnosis, n (%)	
Localized disease	45 (28.3)
*De novo* metastatic disease	93 (58.5)
Unknown stage	23 (14.4)
CNS involvement at diagnosis, n (%)	16 (10.1)
ECOG performance status, n (%)	
0	64 (40.3)
1	38 (23.9)
2	19 (11.9)
3	10 (6.3)
4	4 (2.5)
Immunocompromised patients, n (%)	5 (3.1)
First-line management, n (%)	
Chemotherapy ± immunotherapy	38
Targeted therapy	44
Radiotherapy alone	13
Surgery followed by surveillance	11
Patients lost to follow-up, n (%)	28

At diagnosis, 93 patients (58.5%) presented with *de novo* metastatic disease, including 16 patients (10.1%) with central nervous system (CNS) involvement. Forty-five patients (28.3%) had localized disease, and 23 (14.4%) had an unknown stage, of whom 13 died rapidly with a median survival of only 17 days (range: 1 day–13.8 months). Most patients had preserved performance at presentation (ECOG 0–1 in 64.2%). Five patients (3.1%) were immunocompromised at diagnosis.

Regarding therapeutic management, first-line treatment strategies varied according to disease stage and molecular profile (**Table 1**). Overall, 38 patients received chemotherapy ± immunotherapy, 44 received targeted therapy, and 13 were treated with radiotherapy alone. Eleven patients underwent surgical resection followed by surveillance. Notably, among untreated patients, early mortality remained high, with a median interval between diagnosis and death of 41 days. In addition, 28 patients were lost to follow-up.

### Survival outcomes by treatment modality

**Figure 1:**
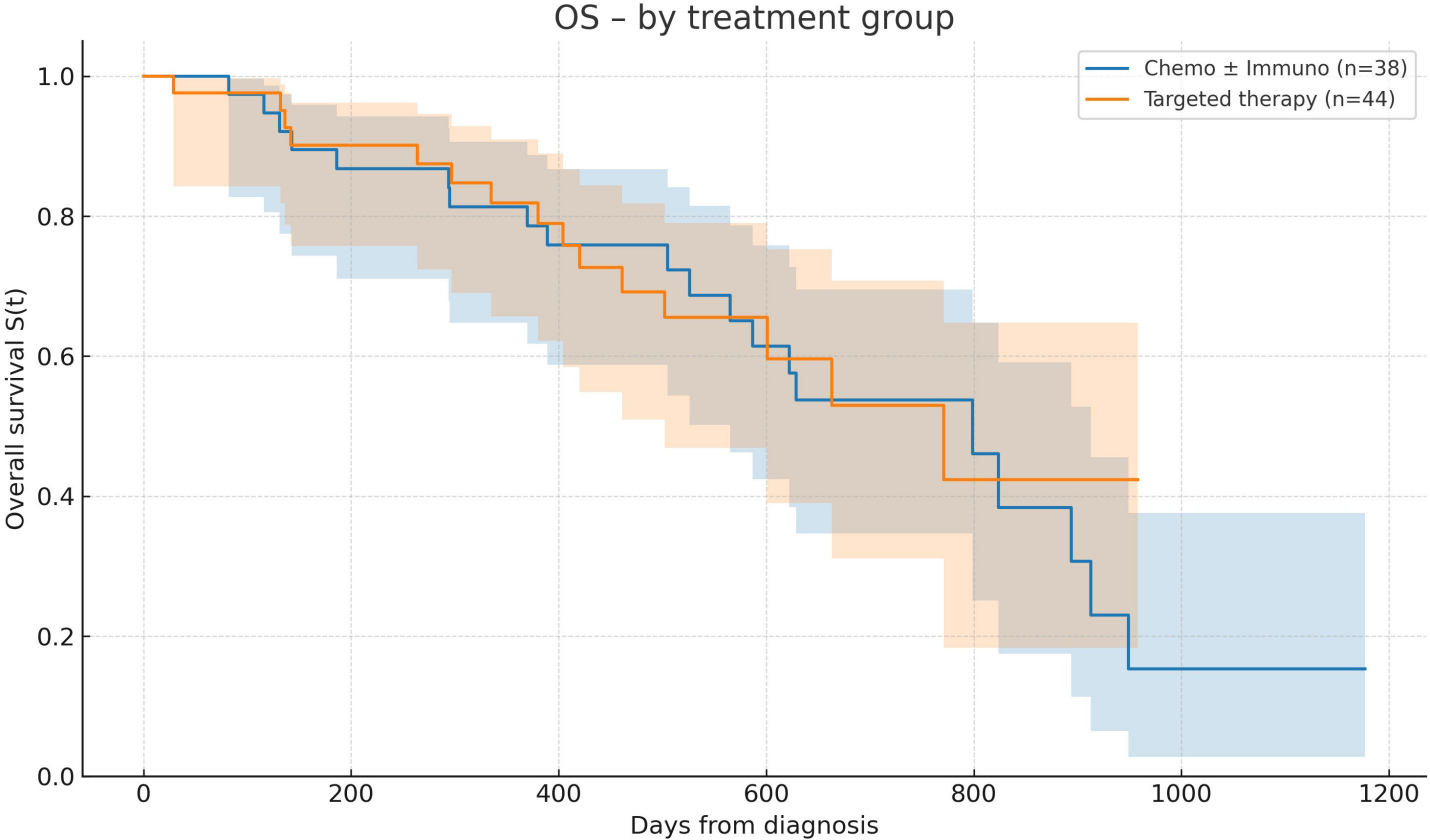
Overall survival according to treatment modality. Kaplan–Meier curves showing overall survival (OS) in patients treated with chemotherapy ± immunotherapy versus targeted therapy. Median OS was 26.3 months in the chemotherapy ± immunotherapy group and 25.3 months in the targeted therapy group, with no significant difference between the two modalities (log-rank p = 0.994).

All survival comparisons between treatment groups are descriptive and unadjusted and should be interpreted as exploratory observations. In the overall cohort, median overall survival (OS) from the date of diagnosis was 19.24 months (95% CI: 12.75–26.2), with OS rates of 67.4% (95% CI: 58.9–74.5%), 61.0% (95% CI: 52.3–68.6%), and 43.9% (95% CI: 34.1–53.2%) at 6, 12, and 24 months, respectively. Among treated patients, those receiving chemotherapy ± immunotherapy (n = 38) achieved a median OS of 26.3 months (95% CI: 18.5–30.0), with OS rates of 89.5% (95% CI: 74.3–95.9%), 81.3% (95% CI: 64.8–90.6%), and 53.8% (95% CI: 34.7–69.5%) at 6, 12, and 24 months. Patients receiving targeted therapy (n = 44) had a median OS of 25.3 months (95% CI: NE–16.5), with OS rates of 90.1% (95% CI: 75.7–96.2%), 81.9% (95% CI: 65.7–91.0%), and 53.0% (95% CI: 31.1–70.8%) at the same timepoints. No statistically significant difference in OS was observed between the two treatment groups (log-rank p = 0.994). Across all treated patients, the median interval between diagnosis and treatment initiation was 2.2 months (mean: 3.4 months, range: 0.2–25.1) (**Figure 1**).

Regarding progression-free survival (PFS), in the treated cohort (n=82), median PFS was 15.3 (95% CI: 0–19.4), with PFS rates of 86.8%, 62.2%, and 21.2% at 6, 12, and 24 months.

**Figure 2:**
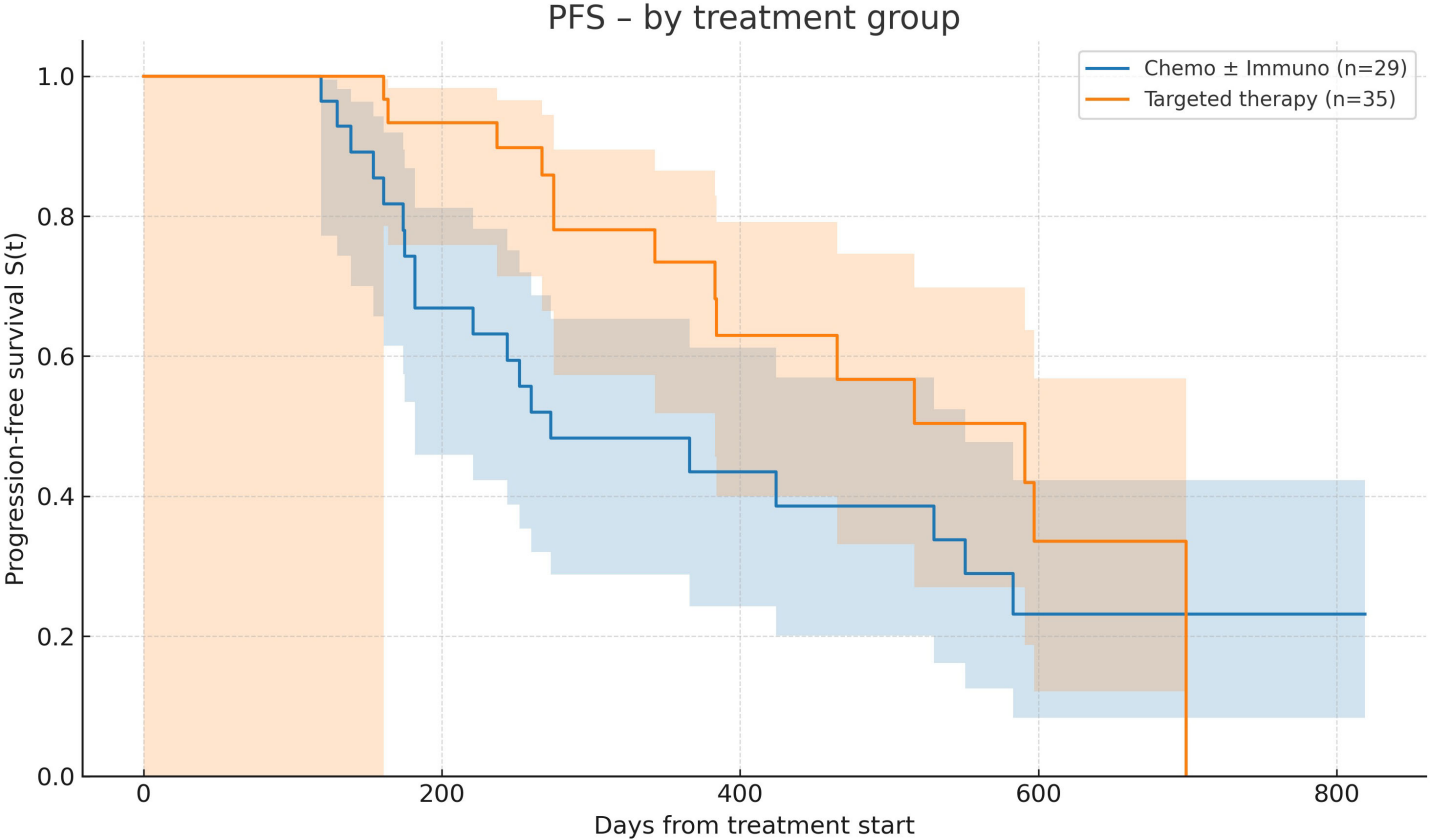
Progression-free survival according to treatment modality. Kaplan–Meier curves showing progression-free survival (PFS) in patients treated with chemotherapy ± immunotherapy versus targeted therapy. Median PFS was 9.0 months for the chemotherapy ± immunotherapy group and 19.4 months for the targeted therapy group. The overall comparison demonstrated a non-significant trend favoring targeted therapy (log-rank p = 0.0528).

By treatment type, among patients receiving chemotherapy ± immunotherapy (n=38), median PFS was 9 months (95% CI: 6–18.1), with PFS rates of 74.3%, 48.3%, and 23.2% at 6, 12, and 24 months. Among patients receiving targeted therapy (n=44), median PFS was 19.4 months (95% CI: 0–23), with PFS rates of 93.3%, 73.4% at 6 and 12 months. No patients remained progression-free at 24 months, resulting in an estimated PFS rate of 0.0% at that time point. The overall comparison showed a non-significant trend favoring targeted therapy (log-rank p = 0.0528) (**Figure 2**).

At the time of analysis, the median follow-up among patients alive at last contact was 15.1 months.

### Molecular profile

**Figure 3:**
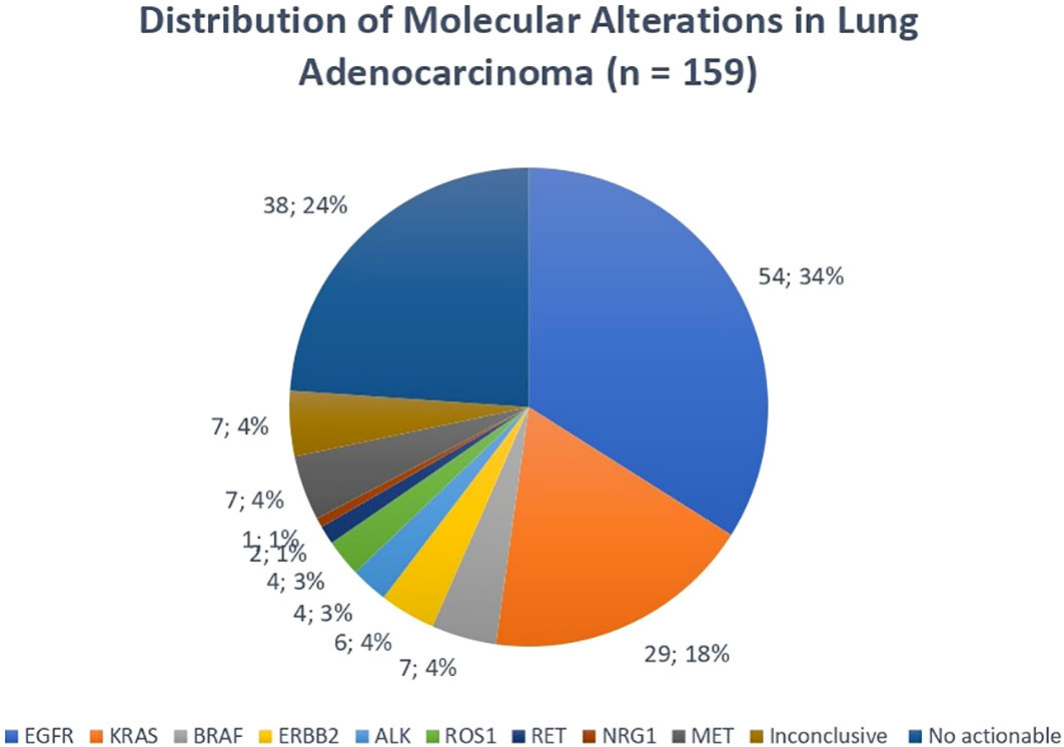
Distribution of molecular alterations in lung adenocarcinoma (n = 159). Pie chart illustrating the relative frequencies of the main oncogenic drivers identified in the cohort, including *EGFR*, *KRAS*, *BRAF*, and *ERBB2* mutations; *ALK*, *ROS1*, *RET*, and *NRG1* gene fusions; and *MET* exon 14 skipping mutations. The proportions of cases without actionable alterations and non-contributive molecular analyses are also shown.

Among the 159 patients with non-small cell lung adenocarcinoma, comprehensive molecular profiling was performed using DNA-based hotspot panel analysis and RNA sequencing. A total of 54 patients (33.9%) harbored an *EGFR* mutation, including 33 “exon 19 deletions”, 16 “L858R substitutions”, 3 “exon 20 insertions”, 2 rare “exon 18 variants”, and 1 T790M mutation acquired after disease progression under a first-line tyrosine kinase inhibitor (TKI). *KRAS* mutations were detected in 29 patients (18.2%), including 8 G12C, 9 G12V, 9 G12D, 1 G12A, and 1 G13C. *BRAF* mutations were identified in 7 patients (4.4%), including 5 V600E variants. Oncogenic exon 20 insertions in *ERBB2* were found in 6 patients (3.7%), all potentially sensitive to trastuzumab deruxtecan. Gene fusions were detected in: 4 *ALK*-rearranged cases, 4 *ROS1*, 2 *RET*, 1 *NRG1* fusion, – and 7 *MET* exon 14 skipping mutations. No actionable molecular alteration was detected in 39 patients (23.89%), and 7 patients (4.4%) had non-contributive molecular results due to insufficient tumor material, mainly related to small bronchoscopic biopsy sample. The overall distribution of molecular alterations is shown in **Figure 3**.

Analysis of the distribution of molecular alterations across these subgroups revealed a statistically significant association between *EGFR* mutations with smoking status (χ²=16.58, p=2.5×10^−4^), with clear enrichment among never-smokers (24/47; 51.1%), and rare in former (0/13) and current smokers (3/21). No statistically significant associations were observed for *KRAS* (p=0.435), *BRAF* (p=0.152), or *ERBB2* (p=0.218), likely reflecting smaller subgroup sizes and limited power.

### PD-L1 expression

PD-L1 expression was available for 132 patients. Tumor proportion scores (TPS) were distributed as follows: 51 patients (38.6%) had a TPS <1 %, 56 patients (42.4%) had intermediate expression (1–49 %), and 25 patients (18.9%) had high PD-L1 expression (≥50 %). PD-L1 status was unknown for 27 patients.

### PD-L1 expression in relation to molecular alterations and treatment response

The distribution of PD-L1 expression across the three TPS categories (<1%, 1–49%, ≥50%) was similar in *EGFR*-mutated and *EGFR*–wild-type tumors, with no significant difference between groups (χ² p = 0.345). *EGFR*-mutated tumors were nonetheless numerically less represented among PD-L1–high cases (TPS ≥50%). Likewise, the distribution of PD-L1 categories did not differ significantly according to *KRAS* mutation status (χ² p = 0.75). The detailed distribution of PD-L1 expression across *EGFR* and *KRAS* subgroups is presented in the **Supplementary Table 1**.

Among patients treated with chemotherapy ± immunotherapy and with available PD-L1 status (n = 34), 12 tumors had PD-L1 TPS <1%, 13 had TPS 1–49%, and 9 had TPS ≥50%. Documented disease progression occurred in 9/12 (75.0%), 6/13 (46.2%), and 5/9 (55.6%) patients in these groups, respectively. Given the limited sample size and the number of patients without documented progression or lost to follow-up, no clear association could be demonstrated between PD-L1 expression and response to chemo-immunotherapy, although tumors with PD-L1 <1% showed a numerically higher rate of progression or lost to follow-up, no clear association could be demonstrated between PD-L1 expression and response to chemotherapy ± immunotherapy. The corresponding contingency table is provided in **Supplementary Table 2**.

## Discussion

This study provides the first comprehensive, real-world characterization of all consecutive cases of lung adenocarcinoma diagnosed in Martinique over an 18-month period, offering an integrated clinical, molecular, and therapeutic overview of a population that remains underrepresented in thoracic oncology research. By capturing disease stage at presentation, genomic alterations, PD-L1 expression, treatment patterns, and real-world outcomes, this work addresses a major gap in evidence for an island population facing structural healthcare inequalities and distinctive environmental and genetic backgrounds. Such descriptive datasets are essential for optimizing diagnostic pathways, accelerating treatment initiation, and improving therapeutic access in regions where delays, early mortality, and limited clinical trial representation remain pressing challenges.

Our cohort is characterized by a strikingly high proportion of patients presenting with *de novo* metastatic lung adenocarcinoma (58.5%) and an additional 14.4% whose stage could not be determined at diagnosis, likely representing patients with advanced disease that remained unclassified due to very rapid clinical decline. More than half of these latter patients died within a median of only 17 days, strongly suggesting undiagnosed metastatic disease with rapid progression. While the proportion of *de novo* metastatic cases in our series appears comparable to the French nationwide KBP-2020-CPHG cohort (57.6%) (15), the excess early mortality observed in the “stage unknown” group indicates that the true burden of metastatic disease in our population may have been underestimated, and could in fact be even higher than reported. Although most patients had a preserved performance status at presentation (ECOG 0–1 in 64.2%), a substantial minority presented with impaired functional status, with approximately one fifth of patients having an ECOG performance status ≥2, a well-established predictor of poor prognosis (14, 16). Within this clinical context, median overall survival (OS) for the entire cohort reached 19.3 months, with OS rates of 67.4%, 61.0%, and 43.9% at 6, 12, and 24 months, respectively. In our cohort, the median interval between diagnosis and treatment initiation was two months. This delay appears relatively long compared with French nationwide data, where a median of 1.6 months (IQR 1–2.4) was reported between diagnostic imaging and treatment initiation (17), and with other series describing shorter intervals of around 1–1.5 months (18). Such delays may have contributed to the early mortality observed in our population. Importantly, 36 patients in the overall cohort died without receiving any systemic therapy, with a median interval of only 1.3 months from diagnosis to death (mean 2 months, range 0–8.8), underscoring the burden of early mortality in untreated patients. Together with the 28 patients lost to follow-up, this subgroup largely contributed to the lower global survival estimates. These findings are consistent with broader observations reported across Caribbean island countries, where late-stage diagnosis, early mortality, and delays in access to cancer care remain major challenges, as highlighted in regional cancer control analyses (19). Our results are considerably better than those reported in large population-based registries, where median OS typically ranges between 10 and 12 months: the UK National Lung Cancer Audit 2022 reported a median survival of ~ 10.7 months with 1-year OS around 48% (20); the French KBP-2020-CPHG study observed 3-month mortality rates of 20% overall and 29% in metastatic NSCLC, with a median OS close to 12 months (15); and SEER data from the United States consistently report median OS around 12 months with 2-year survival of 30–35% (21). However, survival outcomes in our cohort remain below those achieved in pivotal phase III clinical trials in advanced NSCLC. Chemo-immunotherapy combinations have demonstrated median OS of 17–22 months, as shown in KEYNOTE-189 (8, 22) and CheckMate-227 (23). Even longer outcomes have been reported with targeted therapies, such as 38.6 months with first-line osimertinib in EGFR-mutated disease (9) and more than 5 years with alectinib in ALK-positive patients (10, 24).

Within this clinical context, the survival outcomes observed in our cohort can be partly explained by the underlying molecular landscape. Nearly two-thirds of patients harbored an actionable driver alteration, placing our series at the upper range of frequencies reported in contemporary studies using combined DNA and RNA profiling (typically 60–77%) (25, 26). This high prevalence of targetable oncogenic events likely contributed to the relatively favorable OS compared with population-based registries, as approximately one third of our patients received targeted therapies. Of note, the detection of uncommon alterations such as *RET* and *NRG1* fusions underscores the added value of RNA-based fusion assays beyond DNA hotspot panels, maximizing the identification of clinically relevant drivers and expanding eligibility for clinical trials. In contrast, approximately one quarter of patients had no detectable oncogenic driver, representing a subgroup primarily dependent on chemotherapy and/or immunotherapy approaches, and potentially contributing to the poorer outcomes observed in a fraction of the cohort. Importantly, the frequency of *EGFR* mutations in our series (≈33%) is consistent with prior reports describing rates around 36%, further supporting the high prevalence of this alteration in our population (27, 28).

Regarding PD-L1 expression, the distribution observed in our cohort was broadly consistent with international real-world data, although the proportion of tumors with high PD-L1 expression (TPS ≥50%) was slightly lower than the 25–30% usually reported (29). This pattern is consistent with the molecular characteristics of our population, particularly the high prevalence of *EGFR* mutations, as EGFR-driven tumors typically exhibit lower PD-L1 expression and reduced immunogenicity (30). In our exploratory analysis, the distribution of PD-L1 across major oncogenic drivers showed no significant association with either *EGFR* or *KRAS* mutation status (p = 0.345 and p = 0.75, respectively). A trend was nonetheless observed, with *EGFR*-mutated tumors being less frequently represented among PD-L1–high cases (TPS ≥50%). Although not statistically significant, this trend is consistent with previously described patterns and may partly account for the comparatively lower proportion of PD-L1 ≥50% tumors observed in our cohort.

Longer PFS was observed in patients receiving targeted therapies compared with those receiving chemotherapy ± immunotherapy; however, these differences should be interpreted as descriptive and exploratory observations. In patients without actionable mutations receiving chemotherapy ± immunotherapy, the median PFS observed in our cohort was broadly consistent with real-world data (31) and with outcomes reported in pivotal trials (8), supporting the adequacy of treatment response in this subgroup. A numerically better trend was noted in patients with higher PD-L1 expression, although this finding remains exploratory given the limited sample size. However, this advantage did not translate into improved OS, which remained broadly similar across groups. This discrepancy likely reflects the broader range of salvage options available in the chemotherapy ± immunotherapy setting—including sequential immunotherapy and additional chemotherapy lines—whereas patients progressing on tyrosine kinase inhibitors (TKIs) often face limited alternatives in the absence of resistance-directed therapies. Acquired resistance is most frequently driven by secondary mutations, such as EGFR T790M or C797S, *MET* amplification, or resistance variants in *ALK* and *ROS1* (32, 33). Early detection of these alterations, particularly through circulating tumor DNA (ctDNA) analysis, has become a cornerstone of modern treatment strategies, enabling access to next-generation TKIs or rational targeted combinations.

Additionally, in our cohort, *KRAS* mutations were identified in 18% of patients but were not significantly associated with smoking exposure, despite nearly one third of the cohort reporting a smoking history. This observation contrasts with existing literature, where *KRA*S G>T transversions (particularly G12C and G12V) are strongly linked to tobacco-related mutagenesis (34), while *EGFR* mutations are more prevalent among never-smokers (35, 36). The limited sample size of mutation subgroups may account this discrepancy. Previous studies conducted in the French West Indies have highlighted the potential role of occupational exposures related to sugarcane cultivation in lung cancer risk, supporting the relevance of these regional environmental factors (37). These factors may partly account for the divergence observed between our data and international series, and highlight the importance of considering local environmental determinants in addition to classical tobacco-related pathways.

This study has several limitations. First, its retrospective design and the relatively short 18-month study period may limit the generalizability of the findings. This timeframe was deliberately chosen to ensure the feasibility of systematic and comprehensive molecular profiling using both DNA- and RNA-based next-generation sequencing for all included patients, while maintaining financial sustainability. In addition, this period corresponds to the years immediately following the COVID-19 pandemic. Although the impact of the pandemic on diagnostic delays and healthcare-seeking behavior could not be formally assessed, delayed consultations may have contributed to the high proportion of patients presenting with advanced-stage disease at diagnosis. Second, due to the limited sample size and heterogeneity of the cohort, no statistical analysis comparing survival rates was performed. In addition, treatment selection in this real-world setting was strongly driven by baseline clinical characteristics, including molecular profile, disease stage, and performance status, resulting in a high likelihood of confounding by indication. Therefore, survival comparisons should be interpreted cautiously. Comparisons with other French overseas territories such as Guadeloupe or French Guiana would have been highly informative, the absence of comparable clinical and molecular datasets currently limits broader regional analyses. Finally, the absence of resistance profiling in our cohort represents a key limitation, as it precludes estimating the proportion of patients potentially eligible for optimized second-line therapy. For populations such as ours—characterized by a molecular landscape distinct from that reported in Caucasian cohorts and closer to certain Asian series—the integration of ctDNA-based monitoring is especially relevant. Such an approach would improve detection of resistance mechanisms, expand therapeutic sequencing options, and ultimately optimize survival outcomes.

Altogether, this work provides the first integrated description of lung adenocarcinoma in Martinique and highlights the impact of delayed diagnosis, early mortality, and distinctive genomic features on real-world outcomes. These findings underscore the importance of strengthening diagnostic pathways, broadening access to molecular testing, and accelerating treatment initiation to improve lung cancer care in underserved island populations.

## Conclusion

This descriptive study, conducted over an 18-month period, provides a comprehensive overview of the clinical and molecular landscape of lung adenocarcinoma in our population. Nearly two-thirds of patients harbored actionable driver alterations, underscoring the importance of systematic molecular profiling as a core component of therapeutic decision-making. Despite a high prevalence of advanced-stage disease at presentation and a substantial proportion of patients dying before receiving systemic therapy, overall survival in our cohort was comparable to population-based registries, although still inferior to the outcomes reported in randomized clinical trials of advanced NSCLC. These findings highlight persistent challenges in access to care and the absence of dedicated lung cancer screening programs, both of which likely contribute to diagnostic delays and early mortality.

Our results reinforce the need to anchor molecular diagnostics at the center of lung cancer management, including in non-academic or resource-limited settings. Moving forward, the integration of circulating tumor DNA (ctDNA) analysis to detect resistance mechanisms, together with strategies aimed at earlier diagnosis and reducing delays in treatment initiation, will be essential to improving patient outcomes in this unique and underserved population.

## Data Availability

The data supporting the findings of this study are not publicly available due to patient confidentiality and legal restrictions related to health data in France. Requests for access to anonymized data may be directed to the corresponding author and will be evaluated in accordance with institutional and regulatory requirements. Requests to access these datasets should be directed to regine.marlin@chu-martinique.fr

## Supplementary material

Supplementary Table 1 PD-L1 expression distribution according to EGFR and KRAS mutation status.

Supplementary Table 2 PD-L1 expression and disease progression in patients treated with chemotherapy ± immunotherapy.
